# Incidence of Post-Vasectomy Pain: Systematic Review and Meta-Analysis

**DOI:** 10.3390/ijerph17051788

**Published:** 2020-03-10

**Authors:** Austin B. Auyeung, Anas Almejally, Fahad Alsaggar, Frank Doyle

**Affiliations:** Department of Health Psychology, Division of Population Health Sciences, Royal College of Surgeons in Ireland, 123 St. Stephen’s Green, D02 YN77 Dublin, Ireland; ans.mej@gmail.com (A.A.); fahadalmutoutah@rcsi.ie (F.A.); fdoyle4@rcsi.ie (F.D.)

**Keywords:** vasectomy, scalpel, non-scalpel, pain, meta-analysis, systematic review, post-vasectomy pain syndrome

## Abstract

This is the first systematic review and meta-analysis to ascertain incidences of post-vasectomy pain following traditional scalpel, or non-scalpel vasectomy. Electronic databases PubMed, Embase and PsycINFO were searched up to 1 July 2019 for peer-reviewed articles recording post-vasectomy pain. We identified 733 publications, screened 559 after removal of duplicates and excluded 533. Of the remaining 26 full-text articles, 8 were excluded with reasons, leaving 18 for detailed analyses. Meta-analysis was performed on 25 separate datasets (11 scalpel, 11 non-scalpel, 3 other/combined). Study follow-up ranged from 2 weeks to 37 years and sample sizes from 12 to 723 patients. The overall incidence of post-vasectomy pain was 15% (95% CI 9% to 25%). The incidences of post-vasectomy pain following scalpel and non-scalpel techniques were 24% (95% CI 15% to 36%) and 7% (95% CI 4% to 13%), respectively. Post-vasectomy pain syndrome occurred in 5% (95% CI 3% to 8%) of subjects, with similar estimates for both techniques. We conclude that the overall incidence of post-vasectomy pain is greater than previously reported, with three-fold higher rates of pain following traditional scalpel, compared to non-scalpel vasectomy, whereas the incidence of post-vasectomy pain syndrome is similar.

## 1. Introduction

Vasectomy is a form of permanent contraception which is increasingly considered as men age beyond mid-life. The procedure involves sealing a part of the vas deferens, preventing the transport of spermatozoa out of the testis. Vasectomy is achieved in two parts: exposing the vas deferens out of the scrotum (isolation) and blocking the vas (occlusion). Isolation can be done conventionally using a scalpel to make an incision through the scrotum or by a non-scalpel vasectomy (NSV). This involves clamping the vas and overlying skin by an extracutaneous ring and piercing the skin using pointed dissection forceps to gain access. The NSV technique is less invasive, with reduced damage to microvasculature, lymphatics and nerves, unlike the scalpel technique where these microscopic structures are more likely to be severed. Therefore, NSV may result in less trauma and post-procedure pain. As for occlusion, different methods were developed in order to reduce complications. These include excision and ligation, surgical clips, thermal or electrocautery, intraluminal mucosal cautery or chemical occlusion. However, excision and ligation are still the most widely used methods [1].

Complications of vasectomy can be classified as early or late. Early complications include acute pain, haematoma, bleeding, infection and trauma. Late complications are vasectomy failure, fistula formation and chronic pain [2]. Sperm granuloma or congestive epididymitis can present as either early or late complications. Post-vasectomy pain syndrome (PVPS), also known as chronic post-vasectomy pain, is formally defined as an intermittent or constant unilateral or bilateral testicular pain three months or longer in duration which significantly interferes with daily activities of the patient so as to prompt him to seek medical attention [3]. PVPS is a broad term that encompasses different presentations, such as: constant persistent scrotal pain, pain on ejaculation, pain during physical activity, dyspareunia and sensation of fullness of the vas deferens. Assessing chronic pain following vasectomy involves documenting the time of onset, duration, quality of pain, as well as using a visual analogue scale (VAS) to report severity.

Chronic pain following vasectomy is very challenging to diagnose and treat. Being a diagnosis of exclusion, it exposes the patient to a series of investigations and treatment regimens over months. Once a diagnosis is made, the treatment starts with non-invasive behavioural or pharmacological options. If these fail, patients might require invasive surgical interventions, such as repeating the vasectomy with wide excision of the severed ends, microdenervation of the spermatic cord, epididymectomy, vasectomy reversal or orchiectomy [4]. The last resort for patients with debilitating chronic pain is orchiectomy, despite one study by Sweeney et al. [5] stating that 80% of patients who underwent orchiectomy continued to experience pain.

Although chronic post-vasectomy pain is a recognized complication, current literature shows lack of consensus regarding its frequency. Recent narrative reviews report the incidence of post-vasectomy pain to be between 1% and 6% [6,7]. However, these reports were not derived from systematic reviews of the literature, and could therefore be biased. A Cochrane review by Cook et al. [8] details pain outcomes following scalpel versus non-scalpel vasectomy. It is noteworthy that post-vasectomy pain was not the main focus, only two randomized controlled trials were included for analysis, and observational studies were excluded from their review. Further, the two studies differed with respect to pain results, with one of them having a small sample size and high loss to follow-up. Therefore, there is still a significant gap in the literature so the objective of this study is to provide a more accurate picture of the incidence of post-vasectomy pain in contemporary clinical practice, rather than the more restrictive and less generalisable data extracted exclusively from randomized trials. We aim to conduct the first systematic review and meta-analysis pertaining to the incidence of post-vasectomy pain following traditional scalpel, or non-scalpel vasectomy.

## 2. Methods

This review protocol was registered with the International Prospective Register of Systematic Reviews (PROSPERO) on 29 January 2018 in accordance with Preferred Reporting Items for Systematic Review and Meta-Analysis (PRISMA) (registration number: CRD42018087244); https://www.crd.york.ac.uk/prospero/display_record.php?RecordID=87244.

The primary outcome measure was the incidence of post-vasectomy pain, presenting two weeks or later after the procedure, thus ensuring that pain was not related to any post-surgical complication such as infection, haematoma, bleeding or incisional pain. Pain could be assessed using validated pain scales, VAS or patient report. Studies where measurements of complications including pain were reported in men aged 18 years or older, who underwent scalpel or non-scalpel vasectomies, were included in the review and meta-analysis. The secondary outcome measure was the incidence of PVPS.

Electronic databases PubMed, Embase and PsycINFO were searched from inception up to 1 July 2019. Peer-reviewed publications reporting observational studies; cohort, cross-sectional, case control and randomized control trials were eligible for review. Case series, case reports, conference abstracts and articles not published in English were excluded.

The PubMed search strategy used is as follows: (((“Vasectomy”[Mesh]) OR ((((vasectomy[Title/Abstract]) OR vasectomies[Title/Abstract]) OR post-vasectomy[Title/Abstract]) OR post-vasectomies[Title/Abstract]))) AND ((“Pain”[Mesh]) OR pain[Title/Abstract]). No limits, expansions, explosions were applied to the search. The full search histories are provided in Appendix A.

All references were imported into ENDNOTE X8 in preparation for the screening process, and duplicates were identified and removed. All titles and abstracts generated were double screened by two independent reviewers. Any differences after the initial search in terms of inclusion/exclusion, or subsequent data extraction, were settled after discussion between the two reviewers and when necessary, a third reviewer.

Data from included studies were also independently extracted by two reviewers and discrepancies were discussed with a third reviewer if necessary. Extracted information included: author, country, study design, sample size, mean age, inclusion criteria, exclusion criteria, type of procedure, follow-up duration, definition of PVPS, incidence of pain, incidence of PVPS and comments (any aspect of the study that required further commentary/explanation).

For statistical analysis, overall meta-analytic incidence was estimated using the user-written *metaprop_one* command in Stata 15.1, to ensure results that were always within the 1%–100% range [9]. A random effects model was chosen, using the binomial distribution to model the within-study variability, along with "exact" confidence interval estimation for 95% confidence intervals (CIs). Some studies differentiated between mild, moderate and severe pain. To enhance comparability among studies, and to maintain conservative estimates, for overall incidence analysis we used the lowest estimates of any pain with the longest duration of follow-up, with subsequent analyses considering scalpel and NSV separately. The I^2^ test assessed heterogeneity, with >50% indicating "substantial" heterogeneity. Egger’s test was used to determine the potential effects of small study and publication bias.

All studies included for review were assessed using the Cochrane Risk of Bias Tool [10].

## 3. Results

**Figure 1:**
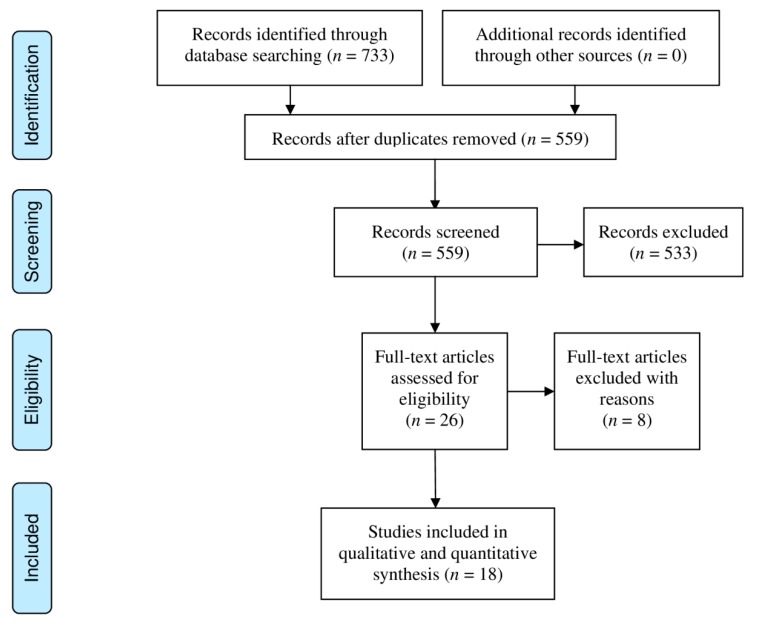
PRISMA flow diagram outlining study selection for incisional scalpel vasectomy and non-scalpel vasectomy.

Figure 1 shows the PRISMA flow diagram for study selection. A total of 733 records were initially identified and 559 records were screened after removal of duplicates. For scalpel and non-scalpel vasectomy, 26 peer reviewed articles remained after exclusion with 18 publications providing estimates for 25 sets of data for meta-analysis (11 scalpel, 11 non-scalpel, 3 other/combined). The earliest study included was published in 1997 by Ahmed et al. [11] and the most recent study was by Sharma et al. in 2014 [12].

Table 1 lists the main characteristics of studies included for review and meta-analysis and Table 2 outlines the major findings on pain in these studies [11,12,13,14,15,16,17,18,19,20,21,22,23,24,25,26,27,28]. These are grouped under Scalpel Vasectomy, Non-Scalpel Vasectomy or Other/Combined Studies, the latter of which included two studies with data from experimental vasectomy procedures that were neither scalpel nor non-scalpel and the other where vasectomy technique was unknown.

All included studies were assessed using Cochrane’s Risk of Bias Tool [10]. Since the majority of studies included in this review used self-reporting questionnaires, this introduces a higher risk of bias (Table 3). Application of the Cochrane bias tool assessment [10] revealed that patients from the included studies were drawn from the same or similar populations, with the exception of Sokal et al. [20], which combined groups of patients from five different countries. In this case, cultural bias could confound the perception or reporting of pain. With Sharma et al. [12], Choe and Kirkemo [14], Leslie et al. [15] and Manikandan et al. [16], we can be confident in the assessment of outcome, whereas there is less certainty for the rest of the included studies, due to lack of standardised or consistent evaluation criteria for pain. Since there are no known associated or prognostic factors linked to chronic post-vasectomy pain, this assessment could not be performed for the included studies. The follow-up of cohorts was adequate for thirteen studies, and was inadequate for five studies, the latter of which could lead to incomplete or missing data. In Bhuyan et al. [23], the post-vasectomy timeline for assessment of pain was unclear. Although the maximum follow-up time was six months post-vasectomy, there was no indication how long the pain lasted and results of the follow-up were not specified.

The primary outcome measured was the incidence of post-vasectomy pain, presenting two weeks or later after the procedure. Pain was measured by patient-reported or investigator-administered questionnaires at the time of follow-up, with the majority utilising some form of graded pain scale. The follow-up durations were widely variable among the studies and ranged from 2 weeks to 10 years (median 1 year) for the scalpel and non-scalpel studies, and up to 37 years for one of the other/combined studies. Sample sizes ranged from 12 to 649 patients.

**Figure 2:**
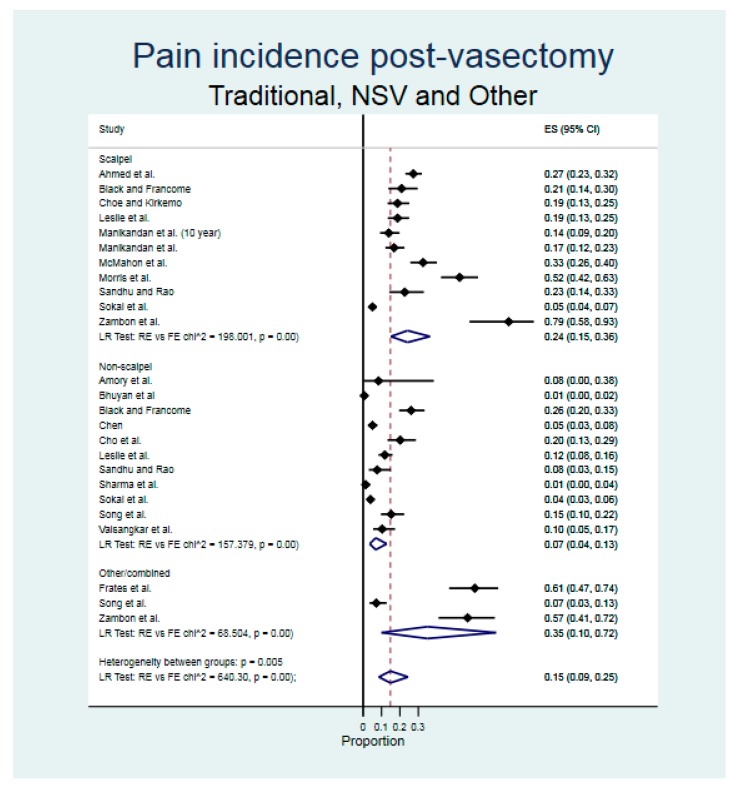
Results of meta-analysis (forest plot) for scalpel and non-scalpel post-vasectomy pain. Legend: ES = effect size, NSV = non-scalpel vasectomy.

The overall incidence of post-vasectomy pain across all studies was 15% (95% CI 9% to 25%), with a higher incidence of 24% (95% CI 15% to 36%) for traditional scalpel vasectomy compared to 7% (95% CI 4% to 13%) for NSV (Figure 2). Heterogeneity among studies was substantial (*p* > 95% for overall estimates and for each subgroup) and there was significant evidence of small study effects or publication bias (β = 0.178, 95% CI 0.079 to 0.277, *p* = 0.001).

**Figure 3:**
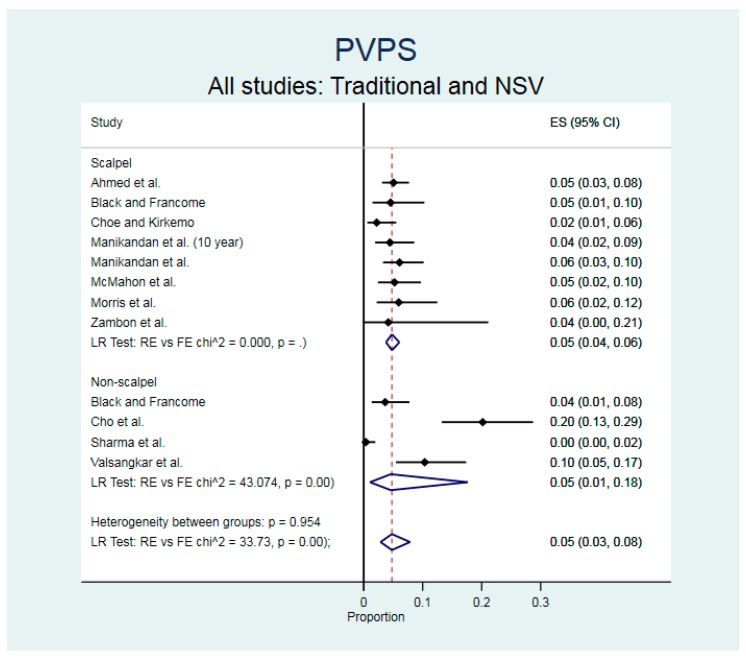
Results of meta-analysis (forest plot) for scalpel and non-scalpel post-vasectomy pain syndrome (PVPS). Legend: ES = effect size, NSV = non-scalpel vasectomy.

The range of PVPS across all studies was 0.4 to 20% (Table 1). The incidence of PVPS was similar for scalpel vasectomy (5% [95% CI 4% to 6%]) and NSV (5% [95% CI 1% to 18%]), albeit there were only 4 NSV studies (Figure 3). Although Davis et al. [3] has provided a formal definition of PVPS, this term has not been strictly adhered to by some other groups [19,21]. Heterogeneity among NSV studies was substantial (*p* > 95% for overall estimates and for each subgroup). In contrast, scalpel vasectomy studies were not heterogeneous. There was significant evidence of small study effects or publication bias for the PVPS estimates (β = 0.061, 95% CI 0.017 to 0.106, *p* = 0.012).

## 4. Discussion

Literature reports have estimated the incidence of post-vasectomy pain to range between 1% to 6% [6,7,8,15,18,29]. In contrast, our results from a systematic review and meta-analysis of all the available literature, show much higher frequencies with the overall average being 15%, following scalpel vasectomy at 24% and after NSV at 7%. However, in studies that reported on both scalpel and non-scalpel techniques, the difference was more modest, with 16.9% pain for scalpel vasectomy compared to 12.3% for NSV. With respect to the other/combined group where studies did not strictly fit into the scalpel or NSV groups, one may have expected that pain levels reported would fall between the scalpel and NSV groups, but it was actually higher than either non-scalpel or scalpel techniques at 35%. This result was due to high pain scores in two of the studies, one of which investigated a failed experimental vas occlusion technique [21] and the other which was an ultrasound study to detect mobile echogenicities [28]. The latter study had a control non-vasectomy group showing no statistical difference in pain compared to the vasectomy group.

Across studies, the incidences of pain following conventional incisional scalpel vasectomy and NSV ranged between 5% to 79% and 0.6% to 26%, respectively. When scalpel, NSV and other/combined studies were included, the overall range of post-vasectomy pain was 0.6% to 79%. These were wide ranges, which resulted in significant heterogeneity, and may have been attributable to the different measures and follow-up durations of the studies. However, there may have been other unmeasured factors that also contributed to these differences. It is noteworthy that the majority of the studies in the NSV group were prospective studies investigating non-scalpel vasectomy techniques, whereas many of the traditional scalpel studies were retrospective, with a focus on chronic pain and complications of vasectomy.

This is the first meta-analysis of the incidence of post-vasectomy pain. We found that the incidence of pain was more than double that reported in previous narrative reviews [6,7]. We also found that the incidence of pain was markedly higher when using the scalpel approach compared to NSV. We included studies that measured chronic pain presenting more than two weeks post-procedure, and were not related to any post-surgical complication such as infection, haematoma, bleeding or incisional pain. There is general agreement in the literature that NSV was a less invasive procedure than traditional incisional vasectomy, the former of which resulted in lower incidences of acute and chronic post-vasectomy pain [8,19,20,30]. The contraceptive success rates of both procedures were similar as no differences in effectiveness were detected between the two approaches [8,13].

There is controversy in the literature regarding the definition of PVPS and subsequently, its reported incidence [31]. Different definitions have been used. Davis et al. [3] and Leslie et al. [15] emphasized intermittent or constant, unilateral or bilateral scrotal pain for a period of more than three months, which interferes with a patient’s daily activities and prompts him to seek medical advice. On the other hand, Sokal et al. [20] and Frates et al. [28] considered that persistent pain at 2 weeks or more post-vasectomy was sufficient to be deemed long-term or chronic. PVPS was not consistently defined across publications and this may have accounted for the wide variation in reported occurrence (0.4% to 20%). A uniform definition for PVPS and its consistent application would help to determine its true incidence and prevalence in the population. Our data suggest that the incidence of PVPS following scalpel and NSV are similar. However, these estimates may not be reliable, due to lack of standardised reporting criteria.

Application of the Cochrane Bias tool [10] revealed strengths and weaknesses in the design of included studies. Some strengths were that within-study patient cohorts were mostly drawn from similar populations, we could be confident in the assessment of exposure, that the outcome of interest was not present at start of the study and that co-interventions were similar between groups. Shortcomings were that we could not be confident in the assessment of outcome for the majority of studies and the follow-up period was inadequate for five of the eighteen studies.

This review has several strengths and limitations. Particular strengths are that the literature was reviewed systematically, risk of bias assessment was used and we followed PRISMA guidelines. We also included all reports of pain, not just PVPS, increasing generalisability. We did not include studies that were not written in English, which may lead to bias. The reporting criteria for pain, and specifically PVPS were not uniformly applied across studies [3,15,20,28], making it difficult to accurately estimate the incidences of different types of post-vasectomy pain. Although we did exclude acute post-surgical complications by not including pain outcomes within 2 weeks of the procedure, some other chronic aetiologies such as testicular atrophy, varicoceles, testicular tumours, spermatoceles etc. were not ruled out. Many of the studies did not indicate whether procedures were performed by urologists, primary care doctors, general surgeons or others, which may have affected outcomes. The majority of included NSV studies were of prospective design, whereas many of the scalpel studies were retrospective. Patients who did not have any complications or pain were probably less likely to be seen in follow up or respond to questionnaires in a retrospective study. This may skew the pain results towards the NSV technique. In spite of this possible bias, we nevertheless observed a greater than three-fold increase in pain with the scalpel technique. Other confounding factors include the wide variation in follow-up duration, high heterogeneity and evidence of study bias among studies included in the analysis. Egger’s test revealed the presence of small study effects, or publication bias due to selective reporting and/or dissemination of research findings. Contributing factors may include biases of language, grey/unpublished literature, publication delays, selective exclusion of negative outcomes or citation bias in favour of positive results.

## 5. Conclusions

The results of our systematic review and meta-analysis indicate that the incidence of post-vasectomy pain is higher than previously reported estimates. Following traditional scalpel vasectomy, the incidence of post-vasectomy pain is more than three-fold higher than after NSV. However, the incidence of PVPS is similar between the two techniques. Therefore, less invasive NSV should be considered as the preferred procedural method compared to the incisional scalpel approach to mitigate the complication of post-vasectomy pain.

## Figures and Tables

**Table 1:** Characteristics of studies included for review.

Study	Sample Size	Mean Age	Follow-Up	Pain %	PVPS %	Pain Scale	Study Design
**Scalpel Vasectomy Studies**							
Ahmed et al. [11]	396	36 years	19 months	27.2%	5.1%	Pain: none, mild, moderate, severe	Retrospective survey
Black and Francome [13]	110	Not recorded	14 weeks	20.9%	4.5%	Pain: none, mild, moderate, excessive	Randomized prospective
Choe and Kirkemo [14]	182	40 years	4.8 years	18.7%	2.2%	VAS for pain (0–10 cm)	Retrospective chart review
Leslie et al. [15]	187	39.9 years	6.8 months	18.8%	Not recorded	VAS for pain (0–10 cm)	Prospective audit
Manikandan et al. [16]	214 180	35.4 years 36.9 years	1 year 10 years	16.8% 13.8%	5.9% 4.3%	VAS for pain (0–10 cm)	Retrospective survey
McMahon et al. [17]	172	34 years	4 years	33%	5%	Discomfort: (1) never, (2) occasional non-troublesome, (3) occasional nuisance, (4) pain affects way of life	Retrospective survey
Morris et al. [18]	101	40.4 years	> 3 years	52%	6%	Discomfort: (1) occasional non-troublesome, (2) occasional nuisance, (3) pain severe enough to seek medical attention	Case-control
Sandhu and Rao [19]	84	30–39 years	Not recorded	22.6%	Not recorded	Pain: moderate to severe	Prospective cohort
Sokal et al. [20]	649	Not recorded	> 2 weeks	5.1%	Not recorded	Pain: none, mild, moderate, severe	Randomized prospective
Zambon et al. [21]	24	Not recorded	12 months	79%	4%	VAS for pain (0–10 cm) and Pain: none, mild, moderate, severe	Prospective cohort
**Non-Scalpel Vasectomy Studies**							
Amory et al. [22]	12	38 years	1 month	8.3%	Not recorded	Five-point Likert scale for pain: none, mild, moderate, extreme, excruciating	Prospective
Bhuyan et al. [23]	649	35 years	6 months	0.6%	Not recorded	Patient reported pain: method unspecified	Prospective
Black and Francome [13]	165	Not recorded	14 weeks	26%	3.6%	Pain: none, mild, moderate, excessive	Randomized prospective
Chen [24]	394	38.9 years	23 months	5%	Not recorded	VAS for pain (0–10 cm)	Randomized prospective
Cho et al. [25]	114	36.3 years	2 months	20.2%	20.2%	VAS for pain (0–10 cm)	Prospective
Leslie et al. [15]	297	39.9 years	6.8 months	11.7%	Not recorded	VAS for pain (0–10 cm)	Prospective audit
Sandhu and Rao [19]	92	30–39 years	Not recorded	7.6%	Not recorded	Pain: moderate to severe	Prospective cohort
Sharma et al. [12]	280	36 years	6 months	1.4%	0.4%	Patient reported pain: method unspecified	Prospective
Sokal et al. [20]	627	Not recorded	>2 weeks	4%	Not recorded	Pain: none, mild, moderate, severe	Randomized prospective
Song et al. [26]	138	31	12 months	15.2%	Not recorded	Pain: (1) at rest, (2) during activity, (3) during coitus	Randomized prospective
Valsangkar et al. [27]	116	44.5	12 months	10.3%	10.3%	Interview: presence or absence of persistent pain	Retrospective case-control
**Other/Combined Vasectomy Studies**							
Frates et al. [28]	56	44.9 years	2 weeks–37 years	60.7%	Not recorded	Pain: present or absent	Prospective cohort
Song et al. [26]	140	31	12 months	7.1%	Not recorded	Pain: (1) at rest, (2) during activity, (3) during coitus	Randomized prospective
Zambon et al. [21]	44	Not recorded	12 months	57%	4%	VAS for pain (0–10 cm); Pain: none, mild, moderate, severe	Prospective cohort

Legend: VAS for pain (0–10 cm)—visual analog scale where a score of 0 represents “no pain” and a score of 100 mm represents “worst imaginable pain”. PVPS—post-vasectomy pain syndrome.

**Table 2:** Major findings on pain in studies included for review.

Study	Major Findings on Pain
**Scalpel Vasectomy Studies**	
Ahmed et al. [11]	Of 396 respondents, 108 patients reported post-vasectomy pain—88 reported brief pain not considered to be chronic while 20 experienced pain for >3 months.
Black and Francome [13]	At 4 weeks post-vasectomy, 43 of 110 respondents reported problems, with 23 reporting continued pain. At 14 weeks post-vasectomy, 5 patients still had excessive pain.
Choe and Kirkemo [14]	At a mean of 4.8 years, 34 of 182 patients reported post-vasectomy scrotal pain, of which 4 were deemed severe enough to adversely affect quality of life.
Leslie et al. [15]	At a mean of 6.8 months after vasectomy, 35 of 187 patients reported continued pain or discomfort.
Manikandan et al. [16]	Of 180 men who had vasectomies 10 years ago, 25 reported new onset pain (severe in 8). Of 214 men vasectomised 1 year ago, 36 reported pain (severe in 13).
McMahon et al. [17]	At 4 years post-vasectomy, 56 of 172 patients had chronic testicular pain or discomfort, with 26 of these cases reported to be troublesome.
Morris et al. [18]	Of men who had a vasectomy >3 years ago, 53 of 101 subjects reported pain or discomfort with 6 experiencing pain severe enough to seek medical advice.
Sandhu and Rao [19]	Of 84 patients who underwent scalpel vasectomy, 19 complained of moderate to severe post-vasectomy pain.
Sokal et al. [20]	Post-vasectomy pain and/or tenderness were reported in 33 of 649 subjects upon long term (>15 days) follow-up.
Zambon et al. [21]	Following traditional scalpel vasectomy, 19 of 24 patients reported mild (7), moderate (11) or severe (1) pain.
**Non-Scalpel Vasectomy Studies**	
Amory et al. [22]	At 1 month post-procedure, 1 of 12 patients reported scrotal pain.
Bhuyan et al. [23]	With 6 months of follow-up, scrotal pain was reported in 4 of 649 patients.
Black and Francome [13]	At 4 weeks post-vasectomy, 54 of 165 respondents reported problems, with 43 reporting continued pain. At 14 weeks post-vasectomy, 6 patients still had excessive pain.
Chen [24]	At a median of 23 months post-vasectomy, 18 of 394 patients reported pain: at rest (5), with activity (12) or with coitus (1).
Cho et al. [25]	At 2 months post-vasectomy, 23 of 114 patients reported new onset scrotal pain which was not correlated with vas obstruction.
Leslie et al. [15]	At a mean of 6.8 months after vasectomy, 30 of 256 patients reported continued pain or discomfort.
Sandhu and Rao [19]	Of 92 patients who underwent non-scalpel vasectomy, 7 complained of moderate to severe post-vasectomy pain.
Sharma et al. [12]	Post-vasectomy scrotal pain was reported in 4 of 280 cases, with one case of chronic pain persisting after 4 months.
Sokal et al. [20]	Post-vasectomy pain and/or tenderness were reported in 25 of 627 subjects upon long term (>15 days) follow-up.
Song et al. [26]	After non-scalpel vasectomy, 21 of 138 patients reported pain during long-term follow-up (within 12 months).
Valsangkar et al. [27]	In patients who had undergone vasectomy in the previous year, 12 of 116 subjects reported persistent scrotal pain.
**Other/Combined Vasectomy Studies**	
Frates et al. [28]	Of 56 patients who had vasectomies (method unspecified) between 2 weeks and 37 years ago, 34 reported scrotal pain.
Song et al. [26]	After insertion of a novel intra-vas device, 21 of 138 patients reported pain during long-term follow-up (within 12 months).
Zambon et al. [21]	After intra-vas injection of material to form a silicone plug, 25 of 44 patients reported mild (17), moderate (7) or severe (1) pain.

**Table 3:** Bias assessment of included studies using Cochrane’s Risk of Bias Tool [10].

Criterion	1	2	3	4	5	6	7	8
**Scalpel Vasectomy Studies**								
Ahmed et al. [11]	Definitely yes	Definitely yes	Probably yes	N/A	N/A	Probably no	Probably yes	Definitely yes
Black and Francome [13]	Definitely yes	Definitely yes	Probably yes	N/A	N/A	Probably no	Probably yes	Definitely yes
Choe and Kirkemo [14]	Definitely yes	Definitely yes	Probably yes	N/A	N/A	Probably yes	Probably yes	Definitely yes
Leslie et al. [15]	Definitely yes	Definitely yes	Definitely yes	N/A	N/A	Definitely yes	Definitely yes	Definitely yes
Manikandan et al. [16]	Definitely yes	Definitely yes	Definitely yes	N/A	N/A	Probably yes	Definitely yes	Definitely yes
McMahon et al. [17]	Definitely yes	Definitely yes	Probably yes	N/A	N/A	Probably no	Definitely yes	Definitely yes
Morris et al. [18]	Definitely yes	Definitely yes	Probably yes	N/A	N/A	Probably no	Definitely yes	Definitely yes
Sandhu and Rao [19]	Definitely yes	Definitely yes	Probably yes	N/A	N/A	Probably no	Probably no	Definitely yes
Sokal et al. [20]	Probably no	Definitely yes	Definitely yes	N/A	N/A	Probably no	Probably no	Definitely yes
Zambon et al. [21]	Definitely yes	Definitely yes	Probably yes	N/A	N/A	Probably no	Probably no	Definitely yes
**Non-Scalpel Vasectomy Studies**								
Amory et al. [22]	Probably yes	Definitely yes	Probably yes	N/A	N/A	Probably no	Definitely yes	Definitely yes
Bhuyan et al. [23]	Definitely yes	Definitely yes	Probably yes	N/A	N/A	Probably no	Definitely no	Definitely yes
Black and Francome [13]	Definitely yes	Definitely yes	Definitely yes	N/A	N/A	Probably no	Probably yes	Definitely yes
Chen [24]	Definitely yes	Definitely yes	Probably yes	N/A	N/A	Probably no	Definitely yes	Definitely yes
Cho et al. [25]	Definitely yes	Definitely yes	Definitely yes	N/A	N/A	Probably no	Definitely yes	Definitely yes
Leslie et al. [15]	Definitely yes	Definitely yes	Definitely yes	N/A	N/A	Definitely yes	Definitely yes	Definitely yes
Sandhu and Rao [19]	Definitely yes	Definitely yes	Probably yes	N/A	N/A	Probably no	Probably no	Definitely yes
Sharma et al. [12]	Definitely yes	Definitely yes	Probably yes	N/A	N/A	Probably yes	Probably no	Definitely yes
Sokal et al. [20]	Probably no	Definitely yes	Definitely yes	N/A	N/A	Probably no	Probably no	Definitely yes
Song et al. [26]	Definitely yes	Definitely yes	Definitely yes	N/A	N/A	Probably no	Definitely yes	Definitely yes
Valsangkar et al. [27]	Definitely yes	Definitely yes	Probably yes	N/A	N/A	Probably no	Probably yes	Definitely yes
**Other/Combined Vasectomy Studies**								
Frates et al. [28]	Definitely yes	Definitely yes	Definitely yes	Mostly yes	Definitely yes	Probably no	Definitely yes	Definitely yes
Song et al. [26]	Definitely yes	Definitely yes	Definitely yes	N/A	N/A	Probably no	Definitely yes	Definitely yes
Zambon et al. [21]	Definitely yes	Definitely yes	Probably yes	N/A	N/A	Probably no	Probably no	Definitely yes

1. Was selection of exposed and non-exposed cohorts drawn from the same population? 2. Can we be confident in the assessment of exposure? 3. Can we be confident that the outcome of interest was not present at start of study? 4. Did the study match exposed and unexposed for all variables that are associated with the outcome of interest or did the statistical analysis adjust for these prognostic variables? 5. Can we be confident in the assessment of the presence or absence of prognostic factors? 6. Can we be confident in the assessment of outcome? 7. Was the follow-up of cohorts adequate? 8. Were co-interventions similar between groups?

## Abbreviations and Acronyms

NSV	non-scalpel vasectomy
PVPS	post-vasectomy pain syndrome
PRISMA	Preferred Reporting Items for Systematic Review and Meta-Analysis
VAS	visual analogue scale

## Appendix A. Search Terms for Various Databases

### Appendix A.1. MEDLINE (OVID)

1. vasectomy/
2. vasectomy.tw.
3. vasectomies.tw.
4. post-vasectomy.tw.
5. post-vasectomies.tw.
6. or/1–5
7. Pain/
8. pain.tw.
9. or/7–8
10. 6 and 9

### Appendix A.2. EMBASE (OVID)

1. Vasectomy/
2. ORCHALGIA.tw.
3. ORCHIALGIA.tw.
4. CHRONIC.tw.
5. SYNDROME.tw.
6. PVPS.tw.
7. chronic testicular pain.tw.
8. CTP.tw.
9. or/2–8
10. 1 and 9

### Appendix A.3. PsycINFO (1806 to Present)

1. Vasectomy/
2. ORCHALGIA.tw.
3. ORCHIALGIA.tw.
4. CHRONIC.tw.
5. SYNDROME.tw.
6. PVPS.tw.
7. chronic testicular pain.tw.
8. CTP.tw.
9. or/2–8
10. 1 and 9
